# Exosomes Derived from Brain Metastatic Breast Cancer Cells Destroy the Blood-Brain Barrier by Carrying lncRNA GS1-600G8.5

**DOI:** 10.1155/2020/7461727

**Published:** 2020-04-06

**Authors:** Yunhe Lu, Lei Chen, Liangdong Li, Yiqun Cao

**Affiliations:** ^1^Department of Neurosurgery, Fudan University Shanghai Cancer Center, Shanghai 200032, China; ^2^Department of Oncology, Shanghai Medical College, Fudan University, Shanghai 200032, China

## Abstract

Brain metastasis is a major cause of death in breast cancer patients. The greatest event for brain metastasis is the breaching of the blood-brain barrier (BBB) by cancer cells. The role of exosomes in cancer metastasis is clear, whereas the role of exosomes in the integrity of the BBB is unknown. Here, we established a highly brain metastatic breast cancer cell line by three cycles of *in vivo* selection. The effect of exosomes on the BBB was evaluated *in vitro* by tracking, transepithelial/transendothelial electrical resistance (TEER), and permeability assays. BBB-associated exosomal long noncoding RNA (lncRNA) was selected from the GEO dataset and verified by real-time PCR, TEER, permeability, and Transwell assays. The cells obtained by the *in vivo* selection showed higher brain metastatic capacity *in vivo* and higher migration and invasion *in vitro* compared to the parental cells. Exosomes from the highly brain metastatic cells were internalized by brain microvascular endothelial cells (BMECs), which reduced TEER and increased permeability of BBB. The exosomes derived from the highly metastatic cells promoted invasion of the breast cancer cells in the BBB model. lncRNA GS1-600G8.5 was highly expressed in the highly brain metastatic cells and their exosomes, as compared to the samples with reduced metastatic behavior. Silencing of GS1-600G8.5 significantly abrogated the BBB destructive effect of exosomes. GS1-600G8.5-deficient exosomes failed to promote the infiltration of cancer cells through the BBB. Furthermore, BMECs treated with GS1-600G8.5-deprived exosomes expressed higher tight junction proteins than those treated with the control exosomes. These data suggest the exosomes derived from highly brain metastatic breast cancer cells might destroy the BBB system and promote the passage of cancer cells across the BBB, by transferring lncRNA GS1-600G8.5.

## 1. Introduction

Breast cancer is the most common cancer in women worldwide and is the second leading cause of cancer death among females [1]. Metastasis is the leading cause of morbidity and mortality in breast cancer patients. Brain metastasis occurs in approximately 15 to 30% of breast cancer patients, with the highest incidence in breast cancer patients with trinegative or basal tumors and Her-2 positive tumors [2, 3]. Although promising advances have recently been made in the treatment of breast cancer, the prognosis of breast cancer patients with brain metastasis remains poor. Therefore, novel insights into the process of brain metastasis in breast cancer are urgently needed.

A vital event in the migration of cancer cells to the brain is the crossing of the blood-brain barrier (BBB). The BBB is comprised of brain microvascular endothelial cells (BMECs), pericytes, astrocytes, endothelial basement membranes, and adjacent neurons [4]. However, how the cancer cells across the BBB to cause brain metastasis is unclear.

Long noncoding RNAs (lncRNAs) are a major focus in cancer research, reflecting their diverse mechanisms in the occurrence and progression of cancer [5]. lncRNAs are at least 200 nucleotides in length and do not encode proteins. Nonetheless, they are vital in various biological processes, including cell differentiation, intracellular homeostasis, genomic imprinting and organogenesis, and various pathological processes [6]. lncRNAs are also important during the metastasis of cancer. For instance, lncRNA AFAP1-AS1 promotes metastasis of nasopharyngeal carcinoma by sponging microRNA- (miR-) 423-5p and regulating the Rho/Rac signaling pathway [7]. MALAT1 lncRNA suppresses lung metastasis of breast cancer cells by binding and inactivating the prometastatic transcriptional enhanced associate domain (TEAD) transcription factor and preventing the binding of TEAD to the target gene promoters [8]. Noncoding RNA activated by DNA damage (NORAD) lncRNA suppresses breast cancer metastasis by sequestering S100P [9]. Moreover, lnc-BM promotes brain metastasis of breast cancer by enhancing the adhesion of cancer cells to cerebral vascular endothelial cells by the JAK2/STAT3 pathway [10]. However, the roles of lncRNA in brain metastasis of breast cancer are still largely unknown.

Exosomes are extracellular vesicles 30 to 100 nm in diameter, which are released into the extracellular environment by different types of cells [11]. A variety of molecules, including miRNAs, mRNAs, and lncRNAs, can be carried within exosomes and transferred from donor cells to recipient cells to trigger phenotypic changes in the tumor microenvironment. Exosomes appear to be important in cancer development, including the formation of premetastatic niches. Exosomes secreted by highly metastatic melanoma cells promote metastatic behavior of the primary tumor by inducing vascular leakage at the premetastatic site and reprogramming bone marrow progenitor cells to a vasogenic phenotype [12]. Exosomal miRNA secreted by hepatocellular carcinoma cells can increase vascular permeability by targeting endothelial connexin to promote tumor lung metastasis [13]. Astrocyte-derived exosomal miR-19a promotes brain metastasis of breast cancer cells by reversibly downregulating the expression of phosphatase and tensin homolog (PTEN) in cancer cells and increasing CCL2 secretion and myeloid recruitment [14]. However, whether exosomal lncRNAs are also involved in the brain metastasis of breast cancer is unknown.

In this study, we aimed to investigate the roles of exosomal lncRNAs in the brain metastasis of breast cancer. A brain metastasis breast cancer cell line was established by injecting immunodeficient female mice with MDA-MB-231-luc-D3H2LN cells, which are highly tumorigenic and metastatic. The effect of exosomal lncRNA on the ability of the cells to cross the BBB was evaluated using an *in vitro model* of BBB.

## 2. Materials and Methods

### 2.1. Establishment of Brain Metastatic Cells

The animal experiments were performed in strict accordance with the Guide for the Care and Use of Laboratory Animals of the National Institutes of Health. This study was approved by the Institutional Animal Care and Use Committee of the Fudan University Shanghai Cancer Center. The brain metastatic cells were established according to a previous study [15]. Female wild-type BALB/c mice with 6 to 8 weeks of age were obtained from Shanghai Ruitamos Biotechnology Co. LTD. (Shanghai, China). A cell suspension containing 2 × 10^5^ MDA-MB-231-luc-D3H2LN breast cancer cells (MDA231) in a volume of 100 *μ*l was injected into the left cardiac ventricle of anesthetized mice. The metastasis of breast tumor cells was monitored by weekly bioluminescence imaging using an IVIS Spectrum device (Caliper Life Science, Hopkinton, MA, USA). After 30 days, brain metastatic tumor cells were dissociated and grown in culture, the generated cell group (brain metastatic cell group 1, MDABR1) was selected in the second and third round *in vivo* selection, yielding MDABR3 cell populations that displayed significantly increased brain metastasis.

### 2.2. Cell Culture

MDA-MB-231-luc-D3H1 cells (Xenogen Co., Alameda, CA) and the obtained MDABR3 cells were cultured in RPMI1640 medium and supplemented with 10% heat-inactivated fetal bovine serum (FBS, Invitrogen, Carlsbad, CA, USA) and antibiotic–antimycotic agents. Human astrocytes (Procell, Wuhan, China) were cultured in DMEM medium containing 10% FBS (Invitrogen) and antibiotic–antimycotic agents. Human pericytes and brain capillary epithelial cells (BMECs, Procell) were cultured in M199 medium containing 10% FBS at 37°C in an atmosphere of 5% CO_2_.

### 2.3. Isolation of Exosomes

Cells were grown to logarithmic phase, and the medium was replaced with a serum-free medium. Culture was continued for 48 h. The culture was centrifuge at 300 *g* for 10 min. The supernatant was collected and dead cells were removed by centrifugation at 2000 *×* *g* for 10 min, followed by centrifugation at 10000 *×* *g* for 30 min to remove cell debris. The supernatant was ultracentrifuged at 4°C, 100000 *×* *g* for 1 h. This step was repeated. The supernatant was removed, and the exosome precipitate was added to precooled 1× phosphate-buffered saline (PBS).

### 2.4. Transmission Electron Microscopy (TEM)

Exosomes were incubated on a carbon-coated copper grid, for 5 min and stained by applying a drop of 2% phosphotungstic acid on the grid for 3 min. Absorbing paper was used to remove the excess liquid, and the grid was air dried for 15 min. The exosomes were observed by TEM.

### 2.5. Transwell Assay

The Transwell assay was used to evaluate cell migration and invasion. Cell migration was assessed using 0.8 *μ*m 24-well chambers (353097, Falcon), and cell invasion was performed using BioCoat™ Matrigel® 0.8 *μ*m 24-well chambers (354480, BioCoat). Cell invasive capacity was determined using inserts coated with Matrigel. Briefly, MDA231 and MDABR3 cells were washed by PBS and released from the wells using trypsin. After washing with the serum-free medium twice, cells were resuspended in the serum-free medium. A total of 500 *μ*l cell suspension (2 × 10^5^ cells/ml) was seeded in the upper chamber, and cells were plated in L-15 medium (Sangon, Shanghai, China) without FBS. The lower chamber contained 700 *μ*l of medium supplemented with 10% FBS. Cells were incubated in 5% CO_2_, at 37°C. After incubation for 24 h, cells in the upper chamber were removed, and cells on the lower surface were stained with 0.1% crystal violet.

### 2.6. Construction of BBB *In Vitro* Model

To investigate the function of exosomes, an *in vitro* model of BBB was established as previously described [16]. In brief, pericytes were plated on the lower surface of the upper chamber and cultured for 24 h. Human BMECs were plated in the upper chamber and cultured to form a uniform monolayer. Astrocytes were cultured in the lower chamber. The BBB *in vitro* model was successfully constructed when the transepithelial/transendothelial electrical resistance (TEER) value exceeded 150 cm^−2^.

### 2.7. Determination of TEER

The barrier function of the BBB model was evaluated by determining its TEER. The resistance values (*O*) were detected using an ERS-2 Voltohmmeter (Millipore, Billerica, MA). The TEER value was calculated by the resistance per unit area [16] as (measurement resistance value − blank resistance value) × 0.33 cm^2^.

### 2.8. Permeability Assay

Exosomes were added to the upper chamber of the BBB model and incubated for 24 h. Cells were washed with PBS twice. The upper chamber received 500 *μ*l rhodamine B isothiocyanate-dextran solution (0.1 mg/ml) composed of a FBS-free medium, and the lower chamber contained 1 ml ECM complete medium and culture for 24 h. The cell culture medium was removed from the upper chamber and the lower chamber and added to a 96-well plate in the dark. Measurements were made using a microplate system at 550 nm and 580 nm, respectively. The permeability rate of each group was calculated as [upper chamber absorbance/time (24 h)^∗^ lower chamber volume (1 cm^3^)]/(upper chamber absorbance^∗^ film area (0.3 cm^2^)).

### 2.9. Invasion Assay

Invasion of MDA231 breast cancer cells was evaluated by the *in vitro* model of BBB. The cells were trypsinized and labeled with green fluorescent protein (GFP). Cancer cells were plated with 2 × 10^4^ cells in a serum-free DMEM and the M199 medium containing 10% serum was used as the chemoattractant in the lower chamber. After 48 h, the noninvading cells were removed and the invading cells labeled with GFP. All assays were performed in triplicate.

### 2.10. Western Blot

Proteins from exosomes and BMECs were extracted, and the concentration was determined using a BCA assay kit (Pierce Biotechnology, Inc., Rockford, IL, USA). Protein samples with 30 *μ*g from each group were separated by 10% sodium dodecyl sulfate-polyacrylamide gel electrophoresis. The resolved proteins were transferred to polyvinylidene fluoride membranes (Millipore, Bedford, MA, USA). The proteins were blocked in 5% milk and incubated with primary antibodies against HSP70 (1 : 2000, #4873, Cell Signaling Technology, Beverly, MA, USA), CD63 (1 : 1000, #sc-365604, Santa Cruz Biotechnology, Santa Cruz, CA, USA), ZO-1 (1 : 1000, #13663, Cell Signaling Technology), claudin-5 (1 : 1000, # sc-374221, Santa Cruz Biotechnology), N-cadherin (1 : 1000, #13116, Cell Signaling Technology), and glyceraldehyde-3-phosphate dehydrogenase (GAPDH, 1 : 1000, #5174, Cell Signaling Technology) overnight at 4°C. The samples were then incubated with anti-rabbit or anti-mouse horseradish peroxidase-conjugated secondary antibody. Enhanced chemiluminescence reagent (Thermo Fisher Scientific, Waltham, MA, USA) was used to show the protein bands and optical density was assessed via an ImageJ software.

### 2.11. Screening for Brain Metastasis-Related lncRNA

The differently expressed lncRNAs between high and low metastatic breast cancer cells were obtained from the data set GSE79540 [10]. The data was downloaded from the GEO database (https://www.ncbi.nlm.nih.gov/geo/query/acc.cgi?acc=GSE79540). The Raw Intensity column was set as the signal value; the seqname was set as ID column. The ID column and the signal value column are deduplicated. Different expressions were analyzed using the DEGseq algorithm, and FDR < 0.05, log2Fold Chang > 1 or <-1 was considered significantly different.

### 2.12. Real-Time PCR

The expressions of lncRNAs were validated by real-time PCR. Total RNA from the exosomes and cells was extracted by TRIzol reagent (Invitrogen). The quantity and quality of the extracted RNA was determined by a NanoDrop 2000 spectrophotometer (Wilmington, DE, USA). The qualified RNA samples with an A260/280 ratio > 1.9 were used for real-time PCR. A PrimeScript RT kit (Takara Bio, Dalian, China) was used to synthesize complementary DNA (cDNA). Real-time PCR was performed using a SYBR-Green PCR kit (Roche Diagnostics, Indianapolis, IN, USA). PCR was performed on an ABI QuantStudio™6 Flex System. U6 was used as the internal reference and the primer sequences used for the PCR are shown in Table 1. The PCR was run at 95°C for 10 min for degeneration, 45 cycles of 95°C for 15 sec, and 60°C for 60 sec and dissociation at 95°C for 10 sec, 60°C for 1 min, and 95°C for 15 sec. The data were analyzed by the 2^−*ΔΔ*Ct^ method. The PCR reactions were all repeated three times.

### 2.13. Statistical Analysis

SPSS 21.0 (IBM, Chicago, IL, USA) was used to analyze the real-time PCR data. Two-tailed Students' *t*-test was used to compare the differences between two groups. Mean value ± standard deviation was used to present the experimental data. *P* < 0.05 was considered statistically significant.

## 3. Results

### 3.1. Establishment of Brain Metastatic Cell Lines

**Figure 1:**
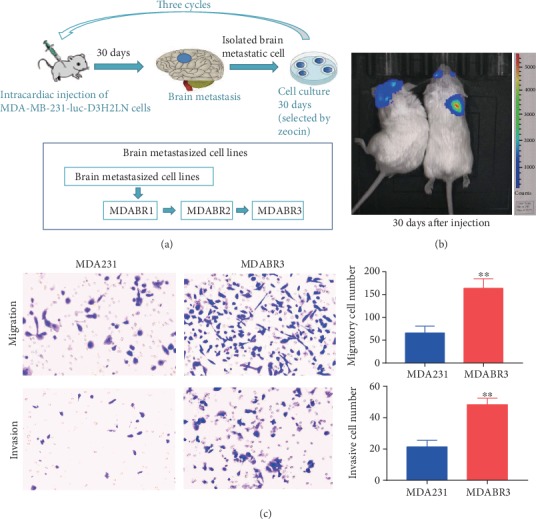
Establishment of breast cancer cell lines with high brain metastatic capacity. (a) Schematic diagram shows the protocol for the *in vivo* selected brain metastatic cells. Breast cancer cells (MDA-MB-231-luc-D3H2LN, MDA231) were injected into the left ventricle of 6–8 week old female wild-type BALB/c mice. After 30 days, biological imaging was performed to observe brain metastases. Cells that had metastasized in the brains of mice were isolated and defined as MDABR1. Half of the MDABR1 cells were frozen, and half were reinjected into mice. After 30 days, biological imaging was performed to observe the brain metastasis, and metastasized cells were isolated and defined as MDABR2. MDABR2 cells were subjected to a third selection to generate MDABR3 (*N* = 10). (b) Bioluminescent images of MDABR3 brain metastases in mice. (c) *In vitro* Transwell assay showing the migration and invasion capacities of MDA231 and MDABR3 cells (*N* = 3, *t*-test, ^∗∗^*P* < 0.01).

To determine the influence of exosomes on the metastatic process of breast cancer, MDA-MB-231-luc-D3H2LN (MDA231) human breast cancer cells were used. The cells have pronounced tumorigenicity and transfer ability and so were used to establish a cell line that readily metastasized to the brain. The MDA231 cells were used for the intracardiac injection into immunodeficiency female mice, and engraftment of cells in the brain was isolated, generating a new high brain metastatic cell line (Figure 1(a)). Brain metastasis was monitored by intraperitoneal injections of luciferin followed by *in vivo* imaging (Figure 1(b)). After dissociating and expanding brain metastatic tumor in culture, the generated cell group 1 (MDABR1) was selected in the second round *in vivo* to produce brain metastatic cell group 2 cells (MDABR2). The MDABR2 cells were subjected to a third round of *in vivo* selection, yielding MDABR3 cell populations that displayed significantly increased brain metastasis. Following ventricle injection, MDA231 cells yielded brain metastases in three of 10 (30%) mice with brain metastases, whereas MDABR3 cells yielded brain metastases in eight of 10 injected mice (80%). *In vitro*, the Transwell assay demonstrated that the migratory and invasive potential of MDABR3 cells was significantly increased compared to that of MDA231 cells (Figure 1(c)).

### 3.2. BBB *In Vitro* Model

**Figure 2:**
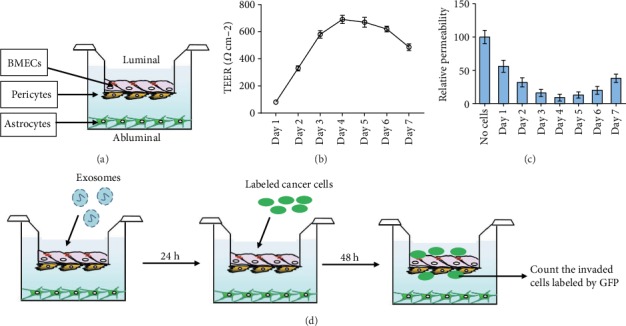
Establishment of the *in vitro* BBB system. (a) Schematic diagram showing the construction of the *in vitro* BBB model, using human brain microvascular endothelium cell (BMEC), brain pericytes, and astrocytes. (b) Transepithelial/transendothelial electrical resistance (TEER) detection in the BBB model. (c) Permeability of the BBB model. The permeability of a cell-free system was used as the control. (d) Schematic diagram displayed the methods for detecting exosome effects on invasion of breast cancer cells through the BBB model.

To investigate the mechanism by which breast cancer cells pass through the BBB, we establish an *in vitro* BBB culture system. *In vivo*, the BBB mainly consists of three types of cells—BMECs, pericytes, and astrocytes—which cooperate with each other to guarantee the function of BBB. Therefore, we constructed an *in vitro* BBB model system in which BMECs were seeded on the upper surface of the upper compartment, pericytes were inoculated on the lower surface of the upper compartment, and astrocytes were cultured in the lower compartment (Figure 2(a)). TEER was used to measure the formation of tight connections between endothelial cells in brain microvessels, which indicated the integrity of the BBB. The TEER of the BBB model was high enough to serve as a model of the BBB *in vivo* (Figure 2(b)). Moreover, the permeability experiment showed that the permeability of the BBB system was very low on days 4 and 5, consistent with the results of TEER (Figure 2(c)). To investigate how exosomes mediated the metastasis of breast cancer cells through the BBB, we incubated the BBB model with exosomes for 24 h, and GFP-labeled breast cancer cells were added to the upper chamber of the BBB model (Figure 2(d)). The effect of exosomes on the BBB was evaluated by counting the GFP-labeled breast cancer cells that successfully crossed the BBB system.

### 3.3. Identification of Exosomes Derived from Breast Cancer Cells

**Figure 3:**
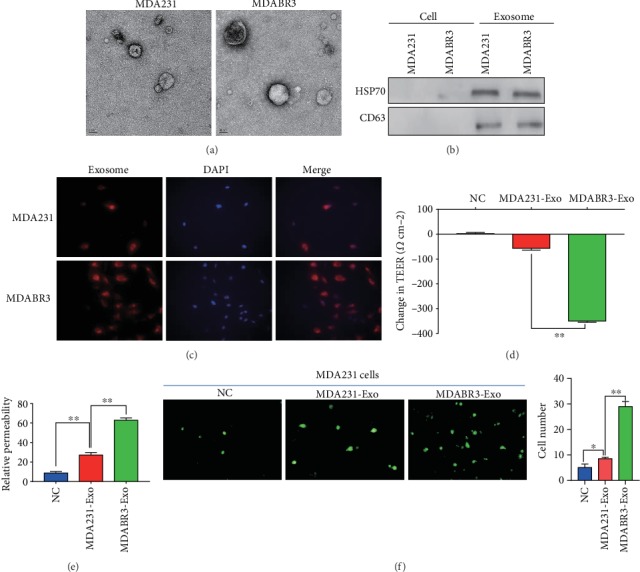
Exosomes derived from high brain metastatic cells destroys BBB permeability. (a) Transmission electron microscopy appearance of exosomes. (b) Western blot measurement of exosome markers in cells and exosomes. (c) Exosomes derived from MDA231 low metastatic breast cancer cells and MDABR3 high brain metastatic breast cancer cells were labeled with PKH26 and added to the BBB model. Representative pictures of BMECs internalizing the exosomes (magnification ×200). (d) Exosomes were added to the BBB system and the TEER value was monitored after 24 h. NC: negative control (BBB system without exosome treatment). (e) Permeability was detected after exosomes incubated BBB system for 24 h. (f) Exosomes were added to the BBB model and incubated for 24 h, MDA231 (PFG-labeled) cells were added to the BBB model, and the number of breast cancer cells crossing the BBB were counted after 48 h. (Magnification ×200, one-way ANOVA, ^∗^*P* < 0.05, ^∗∗^*P* < 0.01).

To evaluate the exosomal roles in brain metastases, we isolated the exosomes from the high brain metastatic MDABR3 cells and low brain metastatic MDA231 cells. TEM and western blot were used to identify the isolated exosomes. TEM revealed that the exosomes were 100 to 150 nm in size with a complete membrane structure (Figure 3(a)). Western blot showed that the exosomes significantly expressed the exosomal markers CD63 and HSP70 (Figure 3(b)). The results suggested the successful isolation of exosomes from breast cancer cells.

### 3.4. Exosomes Derived from High Metastatic Cancer Cells Destroy the BBB

To investigate the influence of exosomes on the BBB system, exosomes were labeled with PKH26 and added to the BBB model. As shown in Figure 3(c), exosomes from all the breast cancer cells, including MDABR3 and MDA231, were incorporated into BMECs. Importantly, the changes in TEER of the BBB model induced by MDABR3 exosomes were significantly higher than the changes induced by MDA231 exosomes (Figure 3(d)). MDABR3 exosomes significantly increased the relative permeability of the BBB model compared to the blank group (Figure 3(e)). Moreover, the permeability of BBB model treated with MDABR3 exosomes was significantly higher than that treated with MDA231 exosomes (Figure 3(e)). Furthermore, to clarify whether the exosomes were sufficient to allow the migration of cancer cells to the parenchymal side of the brain, we determined the extravasation of low metastatic cancer cells posttreating the *in vitro* BBB model with exosomes derived from the MDA231 or MDABR3 cells. As shown in Figure 3(f), low metastatic MDA231 cells could not pass through BBB without the pretreatment of exosomes, whereas the low metastatic MDA231 cells successfully infiltrated to the abluminal side after treating the BBB system with exosomes derived from high metastatic MDABR3 cells. In contrast, exosomes derived from MDA231 cells failed to enhance the migration of low metastatic MDA231 cells through BBB compared with exosomes derived from MDABR3 cells. Taken together, these data indicate that the exosomes secreted from high metastatic breast cancer cells destroy the BBB permeability and thus promote the passage of cancer cells across the BBB.

### 3.5. Exosomes Destroy BBB by Transferring lncRNA GS1-600G8.5

**Figure 4:**
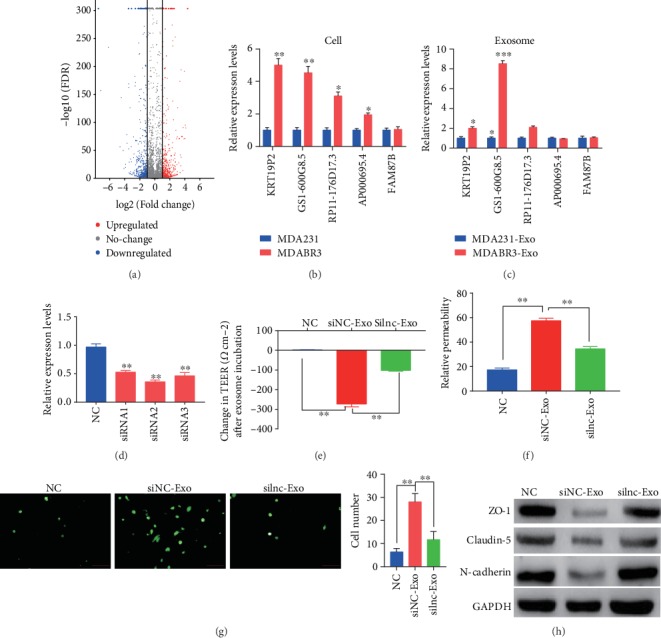
Exosomal lncRNA GS1-600G8.5 promotes BBB passage of breast cancer cells. (a) Volcano plot of differentially expressed lncRNAs between brain metastatic and nonbrain metastatic breast cancer cells. Data were obtained from the GSE79540 database [10]. The red dots represent upregulated lncRNAs and the blue dots represent downregulated lncRNAs in the brain metastatic breast cancer cells compared to the nonbrain metastatic breast cancer cells. (b) The top five upregulated lncRNAs were verified by real-time PCR, in MDABR3 high brain metastatic breast cancer cells and MDA231 parental cells (*N* = 3, *t*-test). (c) The top five upregulated lncRNAs were verified by real-time PCR in exosomes derived from MDABR3 and parental MDA231 cells (*N* = 3, *t*-test). (d) The interference effect of GS1-600G8.5 was verified by real-time PCR in exosomes derived from MDABR3 cells transfected with siRNA. The siRNA-2 with the best interference effect was used for further study. One-way ANOVA. (e) GS1-600G8.5-deficient MDABR3 exosomes and control exosomes were added to the BBB system, and the change in the TEER value was detected after 24 h. One-way ANOVA comparison was done. NC: BBB system without any treatment; siNC-Exo: BBB system treated with exosomes derived from MDABR3 cells that were transfected with siRNA NC; silnc-Exo: BBB system treated with exosomes derived from MDABR3 cells that were transfected with siRNA GS1-600G8.5. (f) Permeability was detected in the BBB system. One-way ANOVA comparison was used. (g) BBB system was treated with exosomes for 24 h, MDA231 (PFG-labeled) cells were added to BBB model, and the number of MDA231 cells crossing the BBB was counted after 48 h. Magnification ×200. One-way ANOVA. (h) Western blot was used to detect the expressions of tight junction and adhesive proteins in the BMECs treated with GS1-600G8.5-deprived exosomes and control exosomes. One-way ANOVA comparison was used; ^∗^*P* < 0.05, ^∗∗^*P* < 0.01, and ^∗∗∗^*P* < 0.001.

To explore the underlying mechanism by which exosomes from high metastatic cells mediated the reconstruction of BBB, we analyzed the lncRNAs differently expressed between high and low metastatic breast cancer cells. From the data set GSE79540 [10], we obtained 1,583 differently expressed lncRNAs, including 654 up- and 929 downregulated lncRNAs, in brain metastatic breast cancer cells compared to parental cells (Figure 4(a)). We selected the top five upregulated lncRNAs and verified their expressions in our high brain metastatic cell lines. Consistently, the expressions of KRT19P2, GS1-600G8.5, RP11-176D17.3, and AP000695.4 were significantly increased in the high brain metastatic MDABR3 cells compared to the low brain metastatic MDA231 cells (Figure 4(b)). Interestingly, only KRT19P2 and GS1-600G8.5 showed upexpression in the exosomes derived from high brain metastatic MDABR3 cells compared to the exosomes from low metastatic MDA231 cells (Figure 4(c)). Moreover, GS1-600G8.5 displayed a higher fold-change compared to KRT19P2. Therefore, we further investigated the effect of GS1-600G8.5 on BBB.

After transfection with GS1-600G8.5 small interfering RNAs (siRNAs), exosome was isolated from the siRNA-transfected MDABR3 cells and generated a GS1-600G8.5-deficient exosome (Figure 4(d)). Moreover, siRNA-2 showed the highest interference effect and was selected for further investigation. As expected, MDABR3 exosomes remarkably increased the changes in the TEER of the BBB model, while the deficiency of GS1-600G8.5 abrogated the effect of MDABR3 exosomes (Figure 4(e)). Similarly, MDABR3 exosomes significantly increased the permeability of the BBB model, whereas silencing of GS1-600G8.5 in MDABR3 exosomes dramatically restored the BBB permeability to the normal level (Figure 4(f)). The low brain metastatic MDA231 cells successfully infiltrated the BBB system by addition with exosomes from NC-transfected MDABR3 cells. However, the exosomes from GS1-600G8.5 siRNA-transfected MDABR3 cells did not facilitate the invasion of low brain metastatic MDA231 cells through the BBB (Figure 4(g)). To further study the mechanism of GS1-600G8.5, we detected the expression of tight junction proteins in BMECs. The expressions of ZO-1, claudin-5, and N-cadherin proteins were significantly decreased in the BMECs treated with MDABR3 exosomes compared to that without treatment (Figure 4(h)). With GS1-600G8.5 deficiency in the exosome, the expressions of ZO-1, Claudin-5, and N-cadherin proteins were increased in the BMECs. These results suggested that exosomes containing lncRNA GS1-600G8.5 may destroy BBB by the tight junction proteins.

## 4. Discussion

Emerging evidences have revealed that exosomes play a crucial role in the metastatic process of cancer [17]. lncRNAs have been shown to be involved in the occurrence and progression of cancer via various mechanisms [18]. However, whether the exosomes modulate the brain metastasis of cancer by carrying lncRNAs remains unknown. In the present study, we successfully established a breast cancer cell line that readily metastasized to the brain. Exosomes derived from these metastatic breast cancer cells could destroy the BBB and thus promote passage of the cancer cells across the BBB. The exosome-facilitated mechanism involved lncRNA GS1-600G8.5.

In the tumor microenvironment, exosomes mediate cancer metastasis mainly by regulating premetastatic niche formation, vascular permeability, macrophage polarization, and tumor-related fibroblasts and other stromal cells. Recently, the role of exosomes in brain metastasis has also attracted attention. Astrocyte-derived exosomes can transfer miRNA-142-3p to lung adenocarcinoma cells and inhibit brain metastasis by suppressing fibroblast growth factor receptor 2 activation [10]. Astrocyte exosomal miR-19a reversibly downregulates the expression of PTEN in breast cancer cells, promoting brain metastasis of breast cancer [14]. Breast cancer cell-derived miR-122 inhibits the glucose uptake of brain astrocytes by downregulating the glycolytic enzyme pyruvate kinase, which promotes brain metastasis [19]. lncRNA XIST knockout promotes the secretion of exosomal miRNA-503 from breast cancer cells, which promotes brain metastasis by mediating microglia cell polarization [20]. The key process for the metastasis of cancer cells to brain is crossing the BBB. How this is done has been unclear. A previous study showed that breast cancer cells release extracellular vesicles containing miR181c to promote brain metastasis by disrupting the BBB [16]. Similarly, our study revealed that lncRNA GS1-600G8.5 containing exosomes derived from breast cancer cells that were prone to undergo brain metastasis disrupted the BBB.

Compared with exosomal miRNA, the role of exosomal lncRNA has been poorly studied. Recently, several exosomal lncRNAs were revealed to be involved in breast cancer. Breast cancer exosomes promote cell proliferation by transferring lncRNA MALAT [21]. Exosome-mediated delivery of lncRNA SNHG14 induces breast cancer chemoresistance to trastuzumab by regulating apoptosis regulators [22]. HOTAIR lncRNA is expressed in exosomes derived from breast cancer patients and is associated with ErbB2/HER2 positivity [23]. Moreover, exosomal lncRNA has been shown to play important roles in the absence of brain metastasis [24]. However, the role of lncRNA shuttled by exosomes in the brain metastasis or breast cancer metastasis has not been reported. In this study, for the first time, we used an *in vitro* system to reveal that high brain metastatic cancer cell-derived exosomal lncRNA GS1-600G8.5 can disrupt the BBB, which promotes the passage of breast cancer cells through the BBB. This data indicates that exosomal lncRNA GS1-600G8.5 might promote brain metastasis of breast cancer. *In vivo* data from an animal model are needed for verification. Furthermore, exosomal lncRNA GS1-600G8 decreased the expression of tight junction proteins, suggesting that exosomal lncRNA GS1-600G8 might disrupt the BBB by targeting tight junction proteins. The mechanism of exosomal lncRNA GS1-600G8.5 remains to be explored in studies that are planned.

Exosomes have been shown to carry various molecules and drugs across the BB in vivo. Exosomes loaded with miR-193b-3p can pass through the BBB and efficiently deliver miR-193b-3p into the hemorrhage region of the brain, which alleviates neuroinflammation in the brains of mice with subarachnoid hemorrhage [25]. Yang et al. showed exosomes loaded with anticancer can across the blood-brain barrier in vivo, which suppressed tumor growth [26]. Exosome-delivered GAPDH-siRNA can pass through the BBB and specifically enter neurons, microglia, and oligodendrocytes in the brain [27]. Exosomes derived from breast cancer cells breach the intact BBB and are taken up by various cells including the brain parenchyma, indicating their capacity to go beyond the BBB in vivo [28]. However, the ability of lncRNA containing exosomes to cross the BBB and the mechanisms involved in this process remain unknown. In the present study, we showed that the GS1-600G8.5 containing exosomes was internalized by BBB in vitro. Based on the above evidences that exosomes carrying various molecules can cross the BBB in brain, we speculate that lncRNAs carried by exosomes might also cross the BBB in vivo. Exosomes have been recommended as novel promising therapeutics and drug delivery vehicles [29]. Our study showed that exosomal lncRNA GS1-600G8.5 can overcome the BBB and contribute to the progression of brain metastasis in vitro, indicating that exosomal lncRNA might act as promising therapeutic targets for brain metastasis in vivo.

In conclusion, lncRNA GS1-600G8.5 was highly expressed in exosomes derived from breast cancer cells that readily metastasized to the brain, compared to the lncRNA in exosomes from low metastatic cells. Moreover, exosomal lncRNA GS1-600G8.5 disrupted the BBB and promoted the passage of breast cancer cells across the BBB, perhaps by targeting tight junction proteins. This data provides new insights into the roles of exosomal lncRNAs in cancer metastasis to the brain.

## Figures and Tables

**Table 1:** Primer and siRNA sequences.

Gene symbol	Primer and siRNA sequence
KRT19P2	F 5′ ACCATTGAGAACGCCAGGATT 3′
	R 5′ ACCTCATCCAGCACCCAAAC 3′

GS1-600G8.5	F 5′ CACAGTGAACCGGACAGTCA 3′
	R 5′ TCAGGCACAATCAGTGGGTC 3′

RP11-176D17.3	F 5′ TGCTCATTCATTCATAAATGCTCTT 3′
	R 5′ AGACGGGAGACAGGAAGTGA 3′

FAM87B	F 5′ TGGGCGTCATTCTTTGTGGT 3′
	R 5′ CCATAGATGAGAGCTGGCCG 3′

AP000695.4	F 5′ TCACAGTGGCCTTGAAGTTCT 3′
	R 5′ CGGGTGTTGTTTGTCAGTGC 3′

GS1-600G8.5 siRNA-1	AUUGUUAACCAUUGCAAAGAA
	CUUUGCAAUGGUUAACAAUAC

GS1-600G8.5 siRNA-2	UCUACUAGUAUUGUUAACCAU
	GGUUAACAAUACUAGUAGAAA

GS1-600G8.5 siRNA-3	UUCUACUAGUAUUGUUAACCA
	GUUAACAAUACUAGUAGAAAC

siRNA NC	UUCUCCGAACGUGUCACGUTT
	ACGUGACACGUUCGGAGAATT

## Data Availability

The data sets used and/or analyzed during the current study are available from the corresponding author on reasonable request.
